# Imatinib-induced Hepatitis in a Patient Treated for Gastrointestinal Stromal Tumor: A Rare Adverse Effect

**DOI:** 10.7759/cureus.2529

**Published:** 2018-04-24

**Authors:** Jamil M Shah, Kyawzaw Lin, Denzil Etienne, Madhavi Reddy, Yingxian Liu

**Affiliations:** 1 Department of Internal Medicine, The Brooklyn Hospital Center, Academic Affiliate of the Icahn School of Medicine at Mount Sinai, Clinical Affiliate of the Mount Sinai Hospital, New York, USA; 2 Division of Gastroenterology and Hepatology, The Brooklyn Hospital Center, Academic Affiliate of the Icahn School of Medicine at Mount Sinai, Clinical Affiliate of the Mount Sinai Hospital, New York, USA; 3 Department of Anatomic Pathology, The Brooklyn Hospital Center, Academic Affiliate of the Icahn School of Medicine at Mount Sinai, Clinical Affiliate of the Mount Sinai Hospital, New York, USA

**Keywords:** drug-induced hepatitis, drug-induced hepatotoxicity, drug-induced liver injury, gastrointestinal stromal tumor, imatinib, gleevec

## Abstract

Gastrointestinal stromal tumors (GISTs) are rare neoplasms of the digestive tract. The clinical behavior of GISTs varies greatly, has extended follow-up, and almost all of the tumors have malignant potential. The introduction of imatinib has led to extraordinary improvements in the treatment of individuals with GISTs (as well as those with Philadelphia chromosome-positive chronic myeloid leukemia (CML) and Philadelphia chromosome-positive acute lymphoblastic leukemia (ALL)). However, there have been notable postmarketing reports of adverse drug reactions of hepatotoxicity with the use of imatinib. By our search, among individuals taking imatinib for the treatment of GIST, only six cases of drug-induced liver injury (DILI) have been reported. Here, we present an interesting case of an elderly woman who developed DILI after taking imatinib for the treatment of GIST. As the liver function tests (LFTs) initially did not improve, it was decided to proceed with an interventional radiology (IR)-guided liver biopsy, which showed a histologic pattern of acute hepatitis, consistent with DILI. Ultimately, discontinuation of the antineoplastic agent led to recovery in the patient’s clinical condition along with normalization of her LFTs over the next several weeks. Thus, it is essential that physicians remain alert for and suspect DILI for any patient being treated with imatinib who presents with a sudden elevation of LFTs. The key to making the diagnosis is stopping the offending agent and closely monitoring the liver enzymes for improvement. When discontinuation of imatinib alone does not lead to improvement in LFTs and the patient’s clinical status, a detailed history should be taken and initial diagnostic testing performed to exclude other etiologies. And, if they are negative, a liver biopsy should be considered.

## Introduction

Gastrointestinal stromal tumors (GISTs), although the most common mesenchymal neoplasms of the digestive tract, are relatively rare primary GI cancers [1]. They originate from the interstitial cells of Cajal, which are cells of the intestinal autonomic nervous system. They function as electrical pacemakers, controlling motility as well as regulating peristalsis [2]. GISTs are identified primarily by the expression of the KIT protein and often carry activating mutations in either the KIT or the platelet-derived growth factor receptor alpha (PDGFRA) genes [1,3]. These neoplasms are frequently discovered in the stomach (40 to 60 percent) and small intestine (30 to 35 percent) [4]. However, they can arise in any part of the digestive tract, from the esophagus to the anus, and infrequently outside the GI tract in the peritoneum, mesentery, and omentum [5].

Individuals with GISTs can present with overt or occult gastrointestinal bleeding. Frequently, they present with nonspecific symptoms, such as vague abdominal pain or discomfort, early satiety, or bloating. Other individuals may be asymptomatic, and the GISTs are detected incidentally during an endoscopic study (where they usually appear as subepithelial masses) or on cross-sectional imaging studies performed for a different reason [6]. These neoplasms often metastasize to the liver and the peritoneum, and infrequently to the regional lymph nodes [5].

The clinical behavior of GISTs varies greatly, has extended follow-up, and almost all of the tumors have malignant potential [7]. The important prognostic factors are tumor size, mitotic rate, and tumor location. Prognostic models such as the tumor, node, and metastasis (TNM) classification system are available to define prognostic groups and approximate tumor aggressiveness.

Prior to 2001, the only available treatment for GISTs was surgery. Furthermore, in roughly half of individuals, complete resection was not possible, and the median survival varied between 10 and 23 months [8]. With the discovery of activating mutations in either the KIT or the PDGFRA genes that promote the growth of these cancer cells, the antineoplastic agent and specific tyrosine kinase inhibitor, imatinib, was developed. Following medication approval in the United States in 2001 as well as full approval for the indication of unresectable and/or metastatic GIST in 2005, the median survival of advanced GIST increased to more than 60 months [9]. Here, we present an interesting case of an elderly woman diagnosed with GIST who developed imatinib-induced liver injury roughly four months after initiation of treatment.

## Case presentation

**Figure 1:**
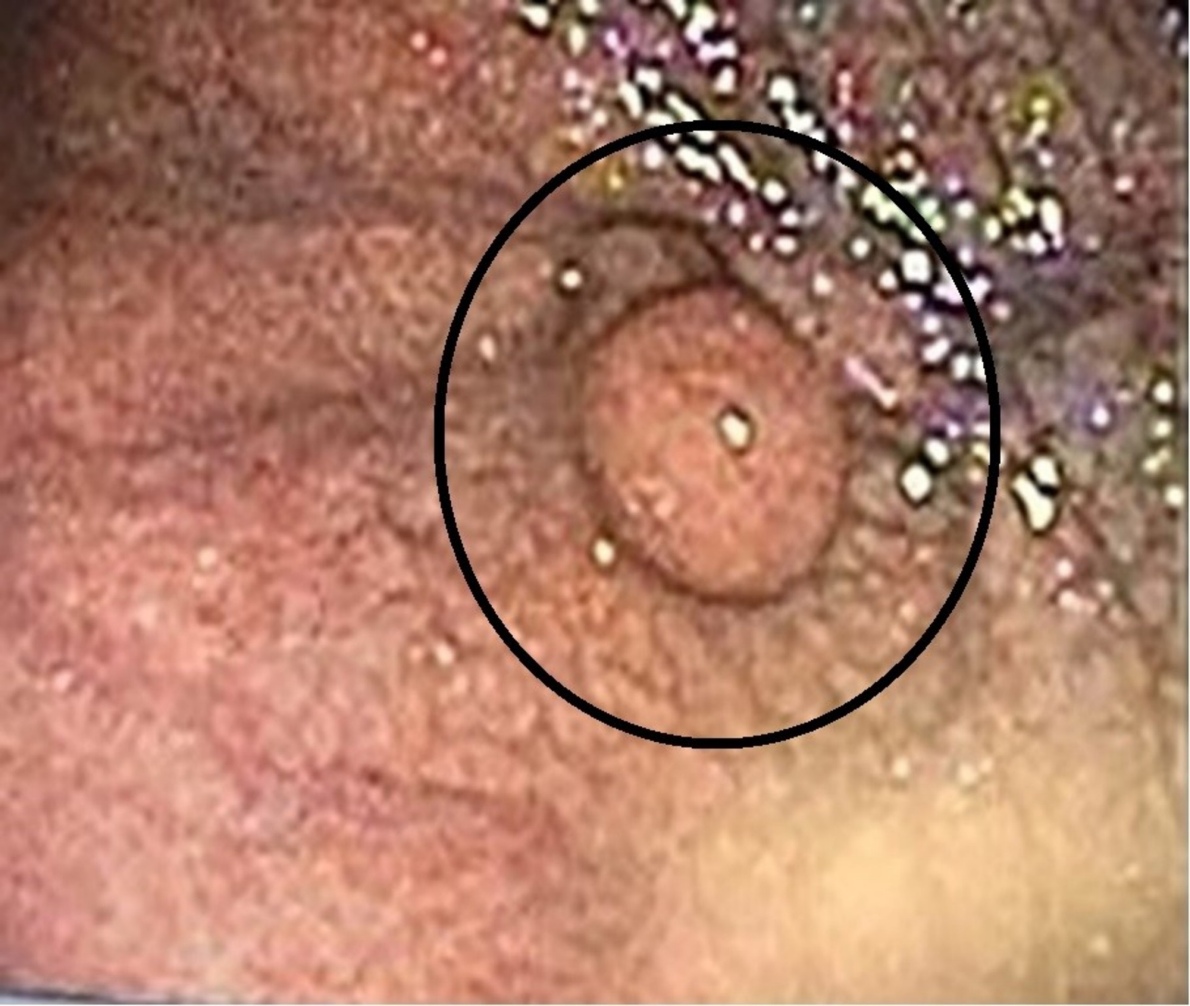
Endoscopic image of the patient's 2.4 cm GIST in the fundus of the stomach, shown with circle. GIST - Gastrointestinal stromal tumor

**Figure 2:**
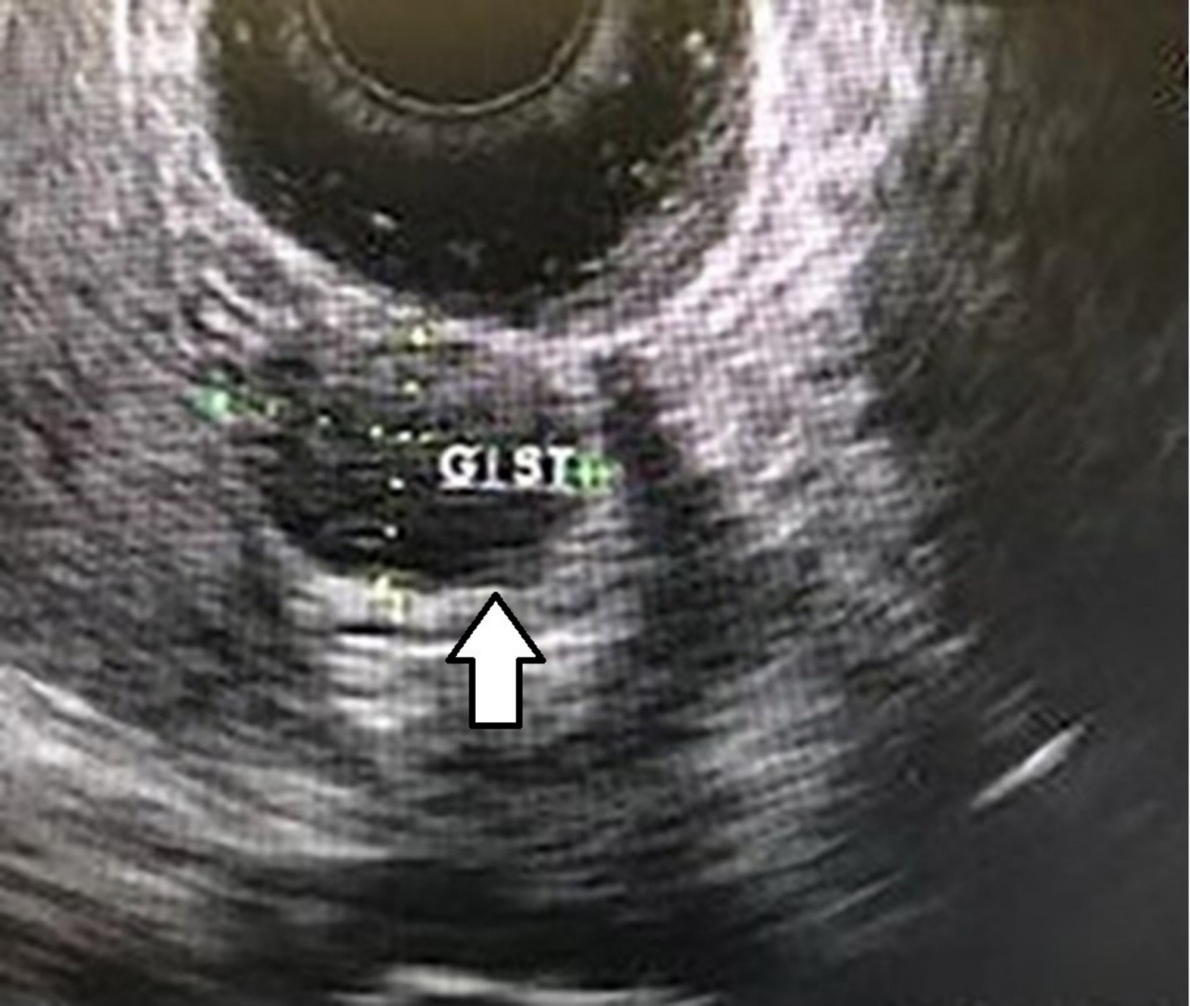
The same 2.4 cm gastric GIST seen on endoscopic ultrasound, shown with arrow. GIST - Gastrointestinal stromal tumor

**Figure 3:**
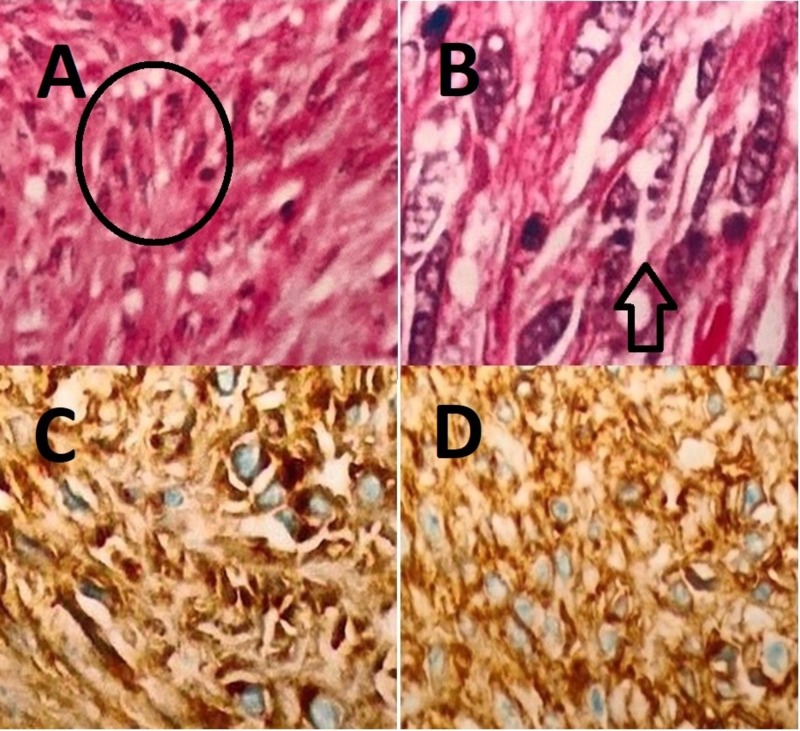
Histopathologic images of the patient’s GIST. A) Atypical spindle cells, shown with circle. H&E stain. 100x. B) Marked atypical spindle cells with polymorphic nuclei, shown with arrow. H&E stain. 400x. C) Positive immunohistochemical staining of CD117 (c-kit). 100x. D) Positive immunohistochemical staining of DOG-1. 100x. GIST - Gastrointestinal stromal tumor.

A 58-year-old Caucasian woman, with past medical history significant for a 2.4 cm GIST diagnosed with esophagogastroduodenoscopy (EGD) (Figures 1, 2) (performed for persistent epigastric pain despite therapy) six months earlier and s/p laparoscopic partial gastrectomy, presented to the emergency department (ED) with new-onset jaundice initially observed by her son four days prior to arrival. Also, she reported generalized weakness, fatigue, and itching for the past several days. The patient reported no previous history of alcohol consumption, intravenous drug use, acquiring tattoos or non-sterile piercings, receiving transfusions of blood or blood products, sexual promiscuity, residence in a developing country, occupational exposure to toxins (she worked as a school teacher), or prior liver diseases. She reported no family history of liver diseases. The earlier biopsy had shown GIST, histologic grade G2 (high grade; mitotic rate > 5/50 per high-power field (HPF)), with spindle cells and no necrosis (Figures 3A, 3B). The margins were negative. The tumor cells were positive / immunoreactive for CD34, CD117, and DOG-1 (Figures 3C, 3D). A recent follow-up computed tomography (CT) scan showed no recurrence.

At presentation, the patient appeared icteric with yellowish discoloration of the skin and sclera. She was afebrile (temperature 97.4) and hemodynamically stable (heart rate 72 beats per minute (BPM), blood pressure 110/85 mm Hg). On physical exam, the abdomen was soft and not distended, with no tenderness and normoactive bowel sounds. Murphy’s sign was negative and there was no guarding nor rigidity.

Initial laboratory testing showed significantly elevated transaminases with aspartate aminotransferase (AST) of 1450 U/L (10-35 U/L) and alanine aminotransferase (ALT) of 1632 U/L (10-55 U/L). Also, there were increased total bilirubin of 4.9 mg/dL (<1.2 mg/dL) and increased direct bilirubin of 2.7 mg/dL (<0.6 mg/dL). The alkaline phosphatase (ALP) was 162 U/L (40-150 U/L), the gamma-glutamyltransferase (GGT) was 95 U/L (5-50 U/L), and the international normalized ratio (INR) was elevated at 1.9 (0.8-1.2). The serum total protein, serum albumin, serum electrolytes, and complete blood count (CBC) were within normal limits. The patient’s Model for End-Stage Liver Disease (MELD) score was calculated to be 28 upon arrival.

Further laboratory testing showed that the serum iron level, total iron-binding capacity (TIBC), serum ferritin, ceruloplasmin level, and thyroid function tests (TFTs) were within normal limits. An acetaminophen level and an autoimmune workup, including antinuclear antibody (ANA), anti-mitochondrial antibody (AMA), and anti-smooth muscle antibody (ASMA), were negative. And, viral serologies for hepatitis B virus (HBV), hepatitis C virus (HCV), cytomegalovirus (CMV), and Epstein-Barr virus (EBV) were negative. An abdominal ultrasound revealed mild hepatomegaly.

**Figure 4:**
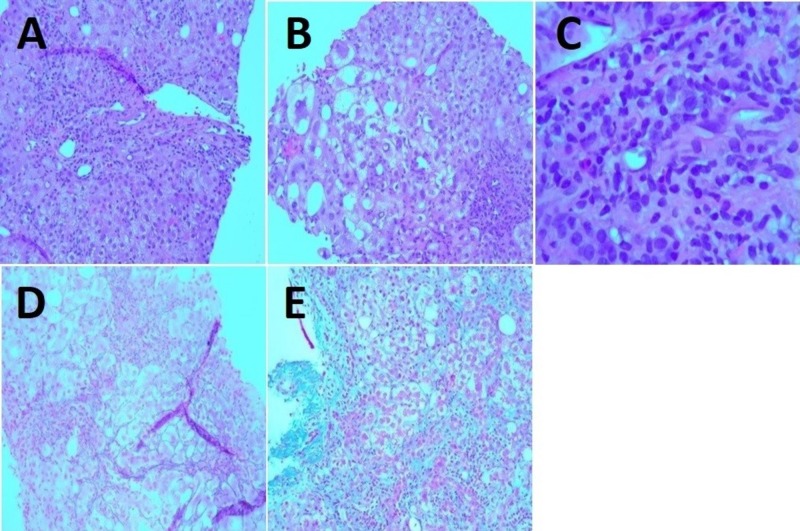
Histopathologic images of the patient’s DILI from liver biopsy. A, B) Moderate steatosis and portal inflammation, as well as ballooning degeneration of hepatocytes indicative of hepatocellular injury. H&E stain. 100x. C) Portal inflammation with a mixed inflammatory cellular infiltrate composed predominantly of lymphocytes and occasional eosinophils. H&E stain. 400x. D) Disarray of liver cell plates with a loss of cohesion between hepatocytes. Reticulin stain. 200x. E) Mild fibrosis. Trichrome stain. 100x. DILI - drug-induced liver injury.

Upon medication reconciliation, it was discovered that the patient was taking imatinib, prescribed by her hematologist-oncologist, for the past four months. The medication was discontinued on hospital admission due to the concern for hepatotoxicity. No other potentially hepatotoxic medications were noted. Prior liver function tests (LFTs), before initiation of imatinib, were all within normal limits. As the LFTs were not improving with conservative management, an interventional radiology (IR)-guided liver biopsy was performed for the purpose of diagnosis as well as to assess the extent of liver injury (Figures 4A-4E).

After seven days, the patient’s transaminases began to decrease, ultimately falling to AST of 483 U/L and ALT of 544 U/L during the hospital admission. The transaminases, as well as the rest of the LFTs, continued to trend down and were within normal range two months later.

## Discussion

Drug-induced liver injury (DILI) is commonly encountered following the use of a variety of medications (both prescription and over-the-counter), herbal medications, and dietary supplements. DILI can be characterized in several ways, including by its clinical presentation (hepatocellular, or cytotoxic, injury; cholestatic injury; or a mixed pattern that involves features of both hepatocellular injury and cholestatic injury), the mechanism of hepatotoxicity (either in a dose-dependent, predictable way or in an unpredictable (idiosyncratic) manner), and the histologic findings (hepatitis, cholestasis, or steatosis) [10]. Usually, DILI is initially described by its clinical presentation. It can then be further characterized by histologic findings if a liver biopsy is performed to make the diagnosis or to evaluate the extent of liver injury.

The diagnosis of DILI is challenging, as it is frequently confounded by incomplete medical information and unrecognized exposures to over-the-counter medications, herbal medications, and toxins. Many individuals with DILI are asymptomatic and are identified only due to laboratory testing showing markedly abnormal LFTs. Others may report nonspecific symptoms, such as right upper quadrant abdominal pain, anorexia, fatigue, malaise, nausea, or pruritus, after starting a medication. Furthermore, drugs can imitate all of the histologic patterns found in primary liver disease, including those of acute, chronic, vascular, or neoplastic disorders caused by other etiologies, thus making the task difficult even on biopsy. The most common histologic pattern of DILI is acute hepatitis, with or without cholestasis. Most cases resolve on discontinuation of the offending medication, but recovery can take months. Infrequently, they can progress to acute liver failure despite medication discontinuation [11].

The introduction of imatinib has led to extraordinary improvements in the treatment of individuals with GISTs (as well as those with Philadelphia chromosome-positive CML and Philadelphia chromosome-positive ALL). However, notable postmarketing adverse drug reactions from the medication have been reported. Elevations in serum transaminases are not uncommon during treatment, but AST or ALT levels more than five times the upper limit of normal appear far less often and should prompt further evaluation [12].

There have been several cases in the literature relating to DILI secondary to imatinib use. In our search we found that among individuals taking imatinib for the treatment of GIST, only six cases of DILI have been reported [13-18]. Usually, the pattern is hepatocellular, although cholestatic patterns have been reported as well. The development of imatinib-induced severe acute hepatitis, such as in our patient above, ranges from within one week of starting the medication to several years out. In the reported cases, discontinuation of the medication proved to be effective. Some case reports have demonstrated a role for immunosuppression with prednisone, as well [19]. Also, there are case reports of reactivation of chronic hepatitis B during treatment with imatinib for the treatment of GIST, which presents in the same way as an acute hepatitis-like syndrome and can be misidentified for DILI [20].

## Conclusions

Physicians, including internists, hepatologists, gastroenterologists, and hematologist-oncologists, should be aware of and suspect DILI for any patient being treated with imatinib who presents with a sudden elevation of LFTs. When prescribing the medication, the liver enzymes should be closely monitored. The key to making the diagnosis is stopping the offending agent followed by improvement in LFTs. When discontinuation of imatinib alone does not lead to improvement in LFTs and the patient’s clinical status, a detailed history should be taken and initial diagnostic testing performed to exclude other etiologies. And, if they are negative, a liver biopsy should be considered.
